# Lessening the erosive influence of electrolyte sports drinks on teeth using L-arginine and aqueous Miswak extract

**DOI:** 10.1186/s13005-025-00560-3

**Published:** 2025-11-29

**Authors:** Hanaa Elgamily, Engie Safwat, Reham Sayed, Samah Mosbah, Ahmed Yossef

**Affiliations:** 1 Restorative and Dental Materials Department, Oral and Dental Research Institutes, National Research Centre, Cairo, Egypt; 2 Dairy Department, Food Industries and Nutrition Research Institute, National Research Centre, Cairo, Egypt; 3 Packaging Materials Department, National Research Centre, Cairo, Egypt; 4 Restorative and Dental Materials Department, Oral and Dental Research Institutes, National Research Centre, 33 El Bohouth St, Dokki, Giza, 12622 Egypt

**Keywords:** Electrolytes, Erosion, L-arginine, Miswak extract

## Abstract

**Objective:**

Excessive intake of electrolyte sports drinks (ESDs) among adolescents and athletes is associated with dental erosion due to their low pH, while their sugar content increases the risk of dental caries. This study aimed to evaluate and compare the protective effects of incorporating either L-arginine or *Salvadora persica* (Miswak) extract into an ESD on enamel surface properties.

**Materials and methods:**

Extracted human premolars were randomly assigned to three groups (*n* = 6): (1) plain ESD, (2) ESD with 1% L-arginine (Arg-ESD), and (3) ESD with 10% *Salvadora persica* extract (Mis-ESD). Each specimen was immersed in its respective solution for 5 min daily over 7 days. Post-treatment assessments included enamel microhardness (VHN), surface roughness (Sa), colour change (ΔE), Ca/P ratio, and scanning electron microscopy (SEM) for surface morphology. A taste acceptability survey was conducted in adult volunteers. Statistical analysis used one-way ANOVA (α = 0.05). In addition, a small panel of adult volunteers (*n* = 20) evaluated the taste acceptability of the modified formulations after providing informed consent.

**Results:**

Both Arg-ESD and Mis-ESD significantly increased enamel microhardness and reduced surface roughness compared to plain ESD (*P* < 0.01), with Mis-ESD showing the greatest improvements. Mis-ESD also enhanced colour stability (*P* < 0.001). SEM images confirmed preservation of enamel structure, particularly in Mis-ESD specimens. Taste testing indicated good palatability for both modified formulations.

**Conclusion:**

Incorporating *Salvadora persica* extract into ESDs significantly improved enamel resistance to erosion and enhanced aesthetic properties. Miswak-enriched ESDs could serve as a preventive option for dental erosion.

**Clinical relevance:**

Formulating ESDs with *Salvadora persica* may reduce their erosive potential while preserving taste acceptability, offering a novel, consumer-friendly strategy to protect enamel in high-risk populations.

## Introduction

The consumption of sports and energy drinks has significantly increased in recent years [1]. Sports drinks are commonly used by athletes to enhance performance, maintain hydration, and replenish electrolytes lost during intense physical activity [2], while energy drinks are intended to boost physical endurance and stimulate metabolism [3]. Although these beverages typically contain high concentrations of carbohydrates such as glucose, fructose, sucrose, and maltodextrins, several studies have reported that their intake is not directly linked to the development of dental caries [4]. Nevertheless, growing concern exists regarding their potential to promote dental demineralization, primarily due to their acidic nature stemming from the presence of citric acid and other acidifying agents [5]. The erosive potential of sports drinks has been estimated to be comparable to that of fruit juices, sweetened beverages, and carbonated soft drinks. Thus, despite the lack of conclusive evidence directly linking energy and sports drinks (ESDs) to caries formation, their potential contribution to enamel erosion and caries risk warrants careful evaluation [6].

The chemical erosive effects of sports and energy drinks on dental tissues—particularly enamel and dentin—continue to attract research attention [7]. Athletes who frequently consume these beverages often experience hyposalivation and xerostomia due to vigorous physical exertion and the resulting fluid loss through perspiration. The reduction in salivary flow impairs the natural buffering, clearance, and remineralizing capacities of saliva, rendering the oral environment more susceptible to acid-induced damage. Consequently, the erosive impact of acidic beverages is amplified in dry oral conditions, which may also compel individuals to consume more of these drinks, further exacerbating the problem [8].

In an effort to address dental erosion non-invasively, various remineralization agents have been investigated [9]. Among these is L-arginine, a semi-essential amino acid that forms part of proteins and peptides and is naturally present in foods such as nuts, dairy products, fish, and meat [10–12]. L-arginine has proven promising in enhancing oral health by inhibiting enamel demineralization, promoting remineralization, alleviating dentinal hypersensitivity, and potentially mitigating alveolar bone loss [13]. Functioning as a prebiotic, L-arginine is metabolized by beneficial oral bacteria via the arginine deaminase system (ADS), leading to ammonia production, which elevates pH levels and helps counteract acid attacks on tooth surfaces [13]. A previous study reported that incorporating 2% arginine into fluoride toothpaste enhanced the remineralization of early enamel lesions and produced a synergistic anti-caries effect [14]. Additionally, Abdeslam et al. in 2022 [15] showed that arginine at concentrations of 2.5% and 8% significantly improved fluoride uptake in demineralized enamel, supporting its use as a therapeutic agent [15]. Furthermore, Elgamily et al. in 2024 [16] demonstrated that 1% L-arginine had notable antimicrobial activity.

Miswak *Salvadora persica* extract, another natural agent, likewise exhibits anticariogenic properties. This plant contains approximately 1.0 µg/g of fluoride as well as substantial amounts of calcium and phosphorus, all of which contribute to its protective role in oral health [17, 18]. Moreover, the slightly bitter taste of its essential oils is known to stimulate salivary flow, enhancing the oral buffering capacity [19]. Previous studies have reported that rinsing with Miswak extract increases plaque pH by stimulating salivary gland secretion, thereby reducing the risk of caries by neutralizing acids produced by cariogenic bacteria [20]. In particular, Hoobi et al. in 2024 [21] found that a 10% aqueous Miswak extract significantly promoted remineralization of early carious lesions and enhanced enamel resistance to acid attacks—surpassing even sodium fluoride in effectiveness.

The erosive potential of ESDs is influenced by several factors, including their pH, the types and concentrations of organic acids used [22]. While removing acidic components entirely could mitigate erosion, such modifications often compromise taste and beverage stability [23]. Similarly, fortifying drinks with calcium or phosphate can help, but changes in flavour and texture may affect consumer acceptance [24–26].

To the best of our knowledge, no previous research has directly compared the protective effects of L-arginine and aqueous Miswak extract against the erosive properties of ESDs, particularly without altering the beverage’s palatability. Thus, the current study aims to explore whether incorporating either L-arginine or aqueous Miswak extract into an ESD formulation can serve as a preventive strategy against enamel erosion. The modified ESDs were evaluated for changes in pH and taste, as well as their impact on enamel surface microhardness, roughness, colour, morphology, and mineral content. The null hypothesis posits that there is no significant difference in the erosive effects between the modified and unmodified ESDs.

## Materials and methods

### Study design

This study involved three experimental groups, each defined by the type of immersion solution used. Group 1, labelled ESD, included specimens immersed in unmodified electrolyte sports drink. Group 2, referred to as Arg-ESD, involved immersion in sports drink supplemented with 1% L-arginine. Group 3, termed Mis-ESD, consisted of specimens immersed in sports drink enriched with 10% aqueous Miswak extract. Extracted human teeth were used in all groups as the substrate for immersion and subsequent analysis. The required sample size per independent treatment group was calculated to be *n* = 6. This follows standard tables for two-group mean comparisons, where a Cohen’s d of 0.8–1.0 typically requires 6–13 specimens per group [27]. Applying this to our three-group design (plain ESD, Arg-ESD, Mis-ESD) yields a total of 18 specimens, which maintains adequate statistical power while meeting practical constraints. The Type I error probability associated with this test of this null hypothesis is (0.05).

### Incorporation of L-arginine and aqueous *Salvadora persica* (Miswak) extract into the Electrolyte Sports Drink (ESD)

A commercially available electrolyte sports drink (LyteSpeed Electrolyte Sports Drink, Building Blox Nutrition, Egypt; batch number 5121/2006) was obtained from a local distributor. According to the manufacturer, each serving (34 g per 500 mL of water) contains sucrose, aerosol 200 (E551), sodium chloride, citric acid anhydrous, potassium dihydrogen phosphate (E340), acacia gum (E414), maltodextrin, orange flavouring, and sunset yellow (E110). The drink was prepared under sterile conditions by dissolving one scoop (34 g) in 500 mL of distilled water in a clean, sealed bottle, followed by vigorous shaking to ensure full dissolution. Three identical preparations of the ESD were made.

L-Arginine powder (MUSCLEADD™ Mega Arginine, Egypt) was also procured locally. Its formulation includes a whey protein matrix composed of whey protein concentrate, isolate, and hydrolysate. Each gram contains 1000 mg of pure L-arginine. To prepare the Arg-ESD solution, 5 g of L-arginine (equivalent to 1% w/v) was added to one of the prepared ESD bottles, thoroughly mixed to ensure uniform dispersion [15, 16].

For the Miswak-modified group, *Salvadora persica* (Miswak) sticks—sourced from a local market (Sewak-Al Falah, Nabq, South Sinai, Egypt; 13 cm in length, Arak tree variety)—were used. The sticks were cut into small pieces and ground into a fine powder using a laboratory blender. To extract the aqueous constituents, 30 g of Miswak powder was soaked in 300 mL of deionized distilled water (Sigma-Aldrich Chemie GmbH, Switzerland) in a sterile, sealed flask and stored at 4 °C for 48 h. The mixture was then centrifuged at 2000 rpm for 10 min, and the supernatant was filtered through Whatman No. 4 filter paper and a 0.45 μm membrane filter (Millipore Corp., Bedford, MA, USA). The filtrate was stored in sterile, screw-capped vials at − 20 °C for 24 h before undergoing lyophilization at − 50 °C and 5 mm Hg pressure using a Snijders Scientific ultra-low temperature freezer (model 85-). The resulting freeze-dried extract was considered 100% pure and stored in an airtight container until use [28]. To prepare the Mis-ESD solution, 50 mg of the freeze-dried Miswak extract (equivalent to 10% w/v) was dissolved in one of the previously prepared ESD bottles and mixed thoroughly [21, 29].

### Measurement of pH

The pH values of all prepared immersion solutions were measured and compared to that of the unmodified electrolyte sports drink (ESD). A digital pH meter (Hanna Instruments HI2211 pH/ORP Meter, Hanna Instruments, Germany) was used for this purpose [30]. For each solution, 50 mL was transferred into a clean, dry glass beaker. Prior to measurement, the pH meter was calibrated using standard buffer solutions with pH values of 4.0 and 7.0. The pH electrode was thoroughly rinsed with distilled water between each measurement to prevent cross-contamination. Each sample was tested in triplicate, and the average of the three readings was recorded to ensure accuracy and reproducibility.

### Taste assessment and sensory evaluation

A duo-trio discrimination test was conducted to evaluate whether participants could perceive a difference in taste between the plain ESD and the modified formulations (Arg-ESD and Mis-ESD) [31]. The test was performed at room temperature (~ 25 °C), a panel of 20 adult staff volunteers (age range 37–45 years) from the Dairy Department (the Food Industry and Nutrition Institute) and the Restorative and Dental Materials Department (oral and dental research institute), National Research Centre, Cairo, (10 panellists from each department) participated. As “occupational groups” may be considered potentially vulnerable, extra care was taken to ensure voluntariness. All participants were fully informed about the study purpose and procedures, participation was entirely voluntary, and written informed consent was obtained prior to tasting. No biological samples, personal identifiers, or health-related data were collected. The study protocol was reviewed and approved by the Medical Research Ethics Committee (MREC) of the National Research Centre, Egypt (Approval No. 03410124/2024).

In the duo-trio test, participants were first presented with a reference sample (the plain ESD), followed by two coded samples—one identical to the reference and the other being a modified ESD. Each participant was asked to identify which of the two coded samples matched the reference in taste. This test was conducted for both the Arg-ESD and Mis-ESD groups, and results were used to assess the distinguishability of the modified formulations from the original.

Regarding Sensory evaluation, a trained sensory panel consisting of 20 evaluators participated in the organoleptic assessment of the three drink formulations: plain ESD, Arg-ESD, and Mis-ESD. Each drink sample (20 mL) was served in identical, odourless white cups coded with random three-digit numbers to prevent bias. No additional information was provided to the panellists regarding the identity of the samples.

All samples were evaluated at room temperature (~ 25 °C), the same temperature at which the products are typically consumed. Each panellist evaluated each formulation in triplicate. A standardized 9-point hedonic scale was used to assess the following attributes: colour, aroma, taste (including acidity, bitterness, and sweetness), and overall acceptability. The scale used was as follows: 9 = excellent, 7 = very good, 5 = good, 3 = fair, and 1 = poor, as previously described by Bergara-Almeida et al. [32].

The sensory evaluation was conducted in a controlled environment to minimize external factors, and the results were statistically analysed to determine the acceptability of the modified formulations compared to the original ESD.

### Preparation of enamel samples and testing procedures

Extracted human premolars were collected from the dental clinic of the Medical and Scientific Centre of Excellence, National Research Centre, Egypt, following the patients’ provision of written informed consent. The study protocol was approved by the Medical Research Ethics Committee (MREC) under approval number 03410124/2024. All teeth were examined under adequate lighting to exclude any specimens with dental caries, enamel hypoplasia, cracks, restorations, or other structural anomalies. Only sound, defect-free teeth were selected for the study.

Selected teeth were ultrasonically cleaned using deionized water to remove debris and organic residues, then stored in artificial saliva at room temperature. The artificial saliva was prepared with the following composition: NaCl (0.400 g/L), KCl (0.400 g/L), CaCl₂·H₂O (0.906 g/L), NaH₂PO₄·2 H₂O (0.690 g/L), Na₂S·9 H₂O (0.005 g/L), and urea (1.0 g/L), with a final pH adjusted to 7.1 [33].

Each tooth was embedded in a self-cure acrylic resin block, leaving the buccal enamel surface exposed. Specimens were randomly allocated into three experimental groups: ESD group (positive control); Immersed in unmodified electrolyte sports drink, Arg-ESD group; Immersed in ESD supplemented with 1% L-arginine, and Mis-ESD group; Immersed in ESD supplemented with 10% *Salvadora persica* (miswak) aqueous extract.

Each tooth specimen was immersed in its assigned solution for 5 min daily over 7 consecutive days [34, 35] at 37 °C in a shaking incubator (SI-100R, HYSC, Korea) set to 30 rpm. The changes of micro hardness, surface roughness, colour, surface micro morphology and mineral loss of the treated enamel’s surface were recorded and were correlated with the changes in immersion solutions’ pH of each group.

### Microhardness testing

The surface microhardness of enamel was measured before and after the immersion regimen using a Vickers microhardness tester (Shimadzu HMV-M, Newage Testing Instruments Inc., USA). A 200 g load was applied for 15 s. Three indentations were made per specimen, and the Vickers Hardness Number (VHN) was calculated as the mean ± standard deviation (SD) of the three readings [36].

### Measurement of surface roughness using AFM

To evaluate changes in enamel surface roughness, atomic force microscopy (AFM) was employed (Anton Paar Tosca™ 200, USA). Surface topography was scanned at randomly selected sites with a 10 μm × 10 μm area using an Arrow NCR tapping cantilever at a 400 × 400-pixel resolution. AFM images were processed using Tosca Analysis Software specialized program according to ISO 25,178 for surface roughness. The roughness parameter evaluated was S_a_ which refers to the arithmetical mean deviation of the roughness [37].

### Colour change assessment

Enamel colour measurements were conducted pre- and post-treatment using the VITA Easyshade^®^ Advance spectrophotometer (VITA Zahnfabrik GmbH, Bad Säckingen, Germany). Colour assessment was recorded following the CIE L*a*b* colour system (the Commission International del’Eclairage L*a* b*). Where, L* represents (lightness from black to white), a* (red to green), b* (yellow to blue), h (hue) and C (chroma). Each measurement was repeated three times, and the average value was recorded. The total colour difference (ΔE) was computed using the following formula:

$$\Delta E_{00}\left(L\ast_1,\alpha\ast_1, b\ast_1;L\ast_2,\alpha\ast_2,b\ast_2\right)=\Delta{E^{12}}_{00}=\Delta E_{00}$$

This formula quantifies perceptible colour change on the enamel surface as a result of erosive or protective effects [38].

### Surface morphology and mineral content analysis

The morphological alterations of the enamel surface samples were observed via scanning electron microscopy (SEM) (Quanta 250 Field Emission Gun, FEI Company, Netherlands), operated at an accelerating voltage of 30 kV. The enamel discs were first sputter-covered with gold in a vacuum evaporator (MED 010; Balzers, Balzers, Liechtenstein), after which microscopically analyzed to gain photomicrographs of the surface morphology of the treated specimens at 1,000× and 2,000× magnification. Representative micrographs were captured at magnifications of 1,000× and 2,000×. Images were obtained at the start of the experiment, and after 7 days of immersion in its assigned solution for 5 min daily. The scanning area by SEM from the same sample was subsequently examined by integrated Energy Dispersive X-ray Spectroscopy (EDX) to quantify changes in mineral content [39]. The EDX point analysis (80 mm^2^, SDD [silicon drift detector], was performed to determine a qualitative elemental analysis of the same specimens (measured elements: Ca, P, C, Mg, O), five points per sample were randomly selected (300 µm^2^ per point), and the mean values were calculated [40].

### Data analysis

Numerical data was represented as mean and standard deviation (SD) values. They were explored for normality and variance homogeneity by viewing the distribution and using Shapiro-Wilk and Levene tests, respectively. pH data were non-parametric and analyzed using Kruskal-Wallis and Dunn’s tests. Roughness data were normally distributed, but with non-homogenous variances, so they were analyzed using Welch one-way ANOVA and Games-Howell tests. Other data were validated for both assumptions and analyzed using one-way ANOVA and Tukey’s tests. Correlations were analyzed using Spearman’s rank-order correlation coefficient. The significance level was set at *P* < 0.05 within all tests. Statistical analysis was performed with R statistical analysis software version 4.4.2 for Windows [41].

## Results

### Changes in pH values for different tested solutions

**Fig. 1:**
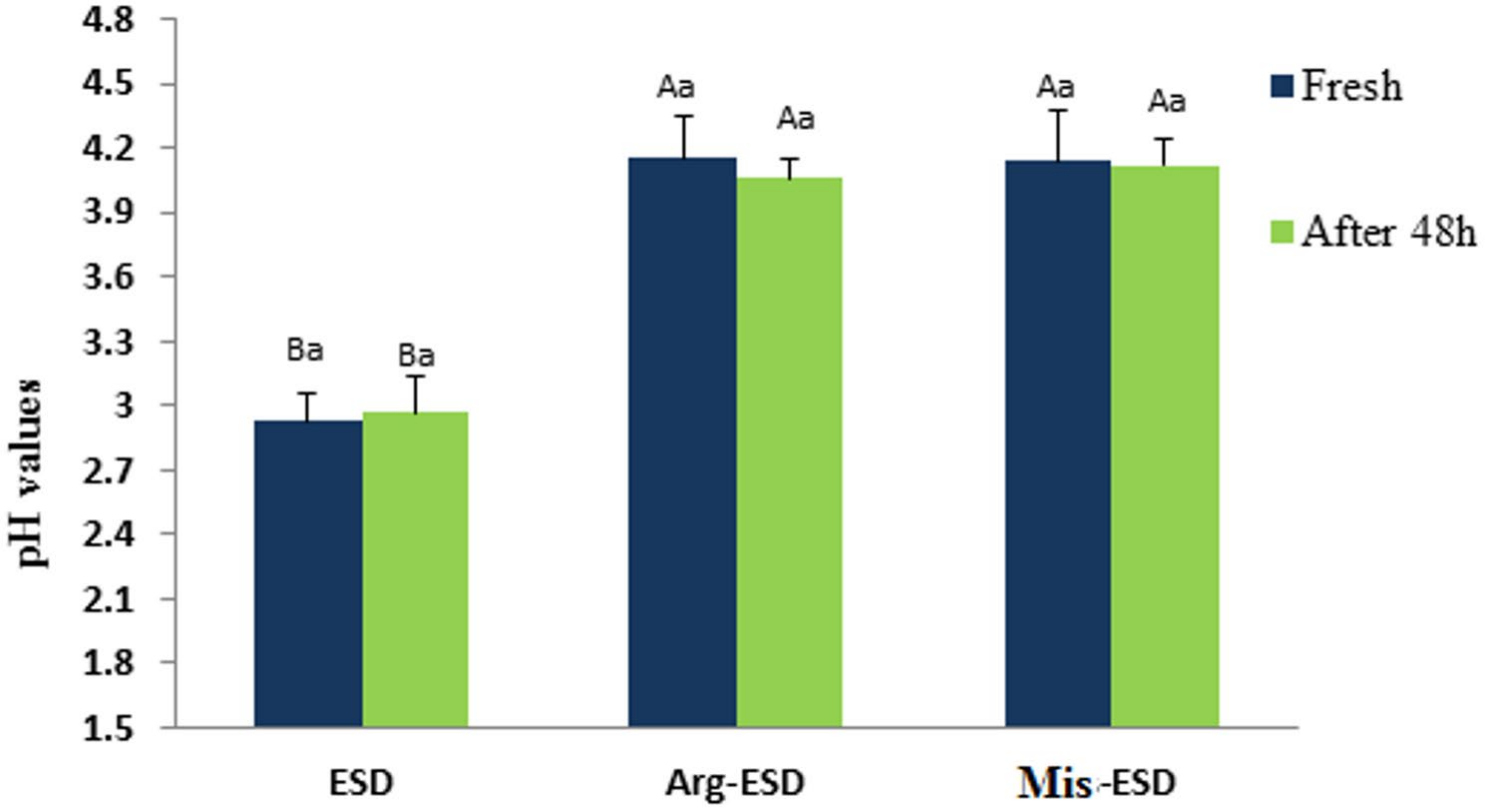
pH values of tested drink solutions in fresh state and after 48 h of cold storage (5 ± 1 °C). Different uppercase letters indicate significant differences between groups at the same time point, while lowercase letters indicate significant differences within groups over time (*p* < 0.05)

Figure 1. illustrates the pH variations for different drink solutions in their fresh condition and after 48 h of cold storage (5 ± 1 °C). All tested solutions had varied levels of pH values. ESD has a fresh pH of 2.93, making it very acidic. The modest increase in pH to 2.97 after 48 h indicates a minor change (*p* > 0.05) in acidity, posing a constant danger of teeth erosion with extended contact. The pH behavior of the Arg-ESD and Mis-ESD formulations was identical, indicating that the addition of arginine or miswak did not significantly (*p* > 0.05) modify the acidity of the drink when compared to each other. Both formulations, Arg-ESD and Mis-ESD, had higher starting pH values (4.15 and 4.14 respectively). After 48 h of storage, both beverages (4.06 for Arg-ESD and 4.12 for Mis-ESD) showed a small reduction in pH, indicating that they stay less acidic (*p* < 0.05) than the ESD solution.

### Results of taste assessment and sensory evaluation

**Fig. 2:**
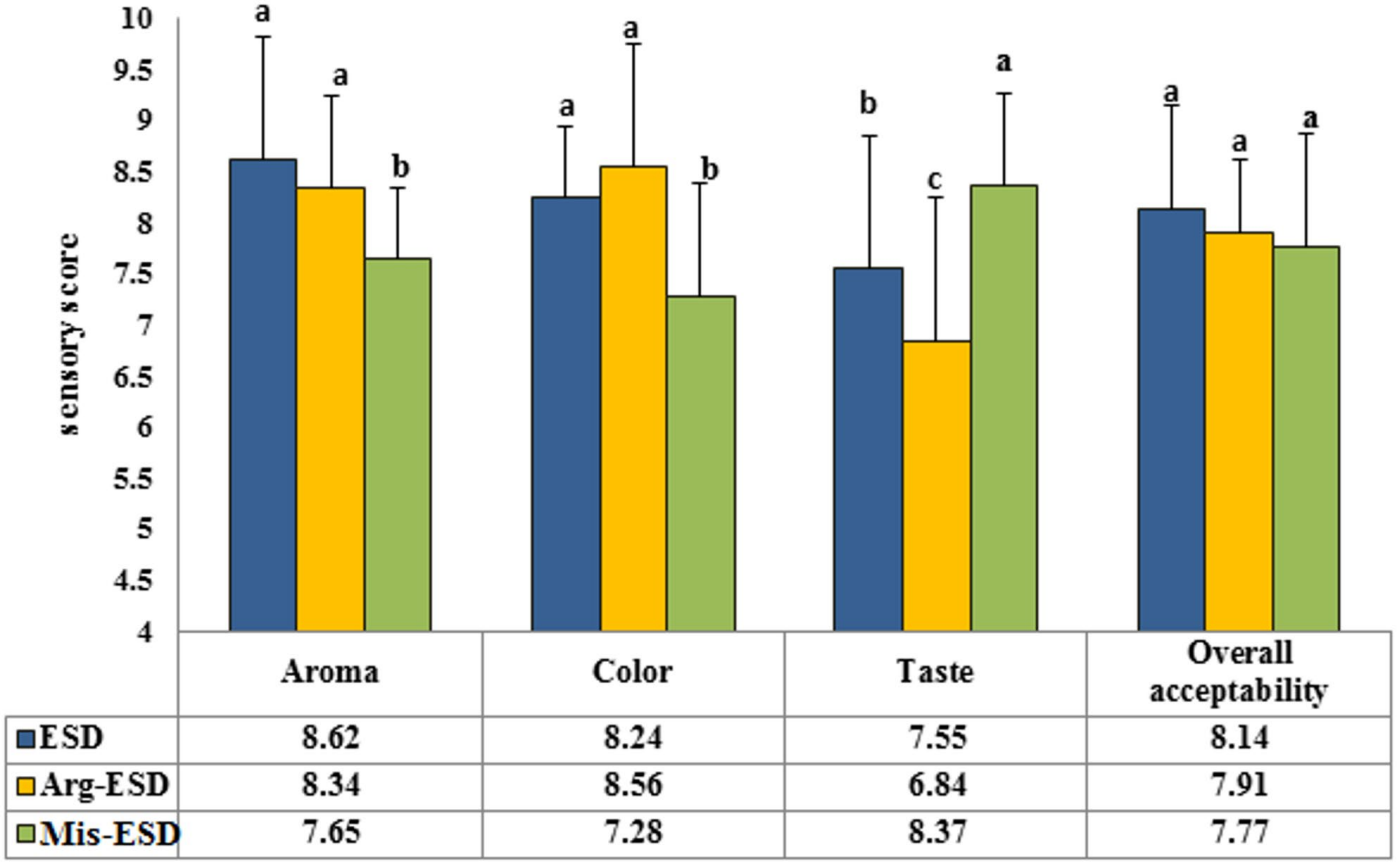
Sensory evaluation scores for ESD (control), Arg-ESD, and Mis-ESD solutions based on aroma, colour, taste, and overall acceptability. Values sharing the same letters are not significantly different (*p* < 0.05)

The sensory properties of the three drink solutions—ESD (control), Arg-ESD, and Mis-ESD—were evaluated in their fresh state, focusing on aroma, colour, taste, and overall acceptability (Fig. 2). Aroma scores were highest for the ESD solution (8.62), indicating that its strong and appealing scent contributed positively to its overall perception. Arg-ESD received a slightly lower aroma score (8.34), while Mis-ESD scored the lowest (7.65), suggesting that the addition of miswak had a modest negative impact on perceived aroma. Colour change Δ*E* was relatively consistent across formulations. Arg-ESD showed the highest colour change (8.56), followed by ESD (8.24), while Mis-ESD showed the lowest colour change (7.28), indicating a less visually appealing appearance. Taste, a critical determinant of consumer preference, varied more distinctly. Mis-ESD received the highest taste score (8.37), outperforming both ESD (7.55) and Arg-ESD, which had the lowest score (6.84). This suggests that the inclusion of miswak may enhance taste perception, while arginine may slightly reduce it. Despite these differences in individual sensory attributes, no statistically significant difference (*p* > 0.05) was found in overall acceptability among the three drinks. All formulations received comparable scores, indicating that modifications with arginine or miswak did not adversely affect consumer acceptance.

### Results of hardness, roughness, lightness, colour, and pH changes

Table 1 presents the intergroup comparisons of hardness change, surface roughness, lightness change (ΔL), overall colour change (ΔE), and pH change. Statistically significant differences were observed across all groups, (*P* < 0.001) for all, except pH change at *P* = 0.023).

**Table 1:** Intergroup comparisons of hardness, roughness, lightness, colour, and pH change

Measurement	Mean ± SD			Tests statistic	*p*-value
	Arg-ESD	Mis-ESD	ESD		
Hardness change	76.15 ± 7.84^B^	109.67 ± 17.67^A^	36.22 ± 12.28^C^	46.40	< 0.001*
Roughness change (µm)	0.07 ± 0.02^B^	0.05 ± 0.00^B^	0.17 ± 0.02^A^	60.84	< 0.001*
Lightness change (ΔL)	6.63 ± 2.18^B^	12.07 ± 1.79^A^	6.48 ± 1.68^B^	16.91	< 0.001*
Colour change (ΔE)	5.65 ± 0.58^B^	10.64 ± 0.37^A^	5.79 ± 0.66^B^	161.18	< 0.001*
pH change	0.10 ± 0.01^A^	0.02 ± 0.00^AB^	−0.04 ± 0.01^B^	7.51	0.023*

Values with different superscripts letters within the same horizontal row indicate statistically significant differences * (*p* < 0.05)

In terms of hardness change, the Mis-ESD group demonstrated the highest mean increase (109.67 ± 17.67), followed by Arg-ESD (76.15 ± 7.84), with the ESD group showing the lowest (36.22 ± 12.28). All pairwise differences were statistically significant. For surface roughness (S_a_), Both Arg-ESD (0.07 ± 0.02 μm) and Mis-ESD (0.05 ± 0.00 μm) groups showed a significantly lower surface roughness compared to ESD (0.17 ± 0.02 μm) group. The lightness change (ΔL) was significantly higher in the Mis-ESD group (12.07 ± 1.79) than in Arg-ESD (6.63 ± 2.18) and ESD (6.48 ± 1.68) groups. Similarly, colour change (ΔE) was most pronounced in the Mis-ESD group (10.64 ± 0.37), significantly higher than both the Arg-ESD (5.65 ± 0.58) and ESD (5.79 ± 0.66) groups. Lastly, pH change was significantly greater in the Arg-ESD group (0.10 ± 0.01) compared to ESD (−0.04 ± 0.01), with Mis-ESD showing an intermediate value (0.02 ± 0.00).

Spearman’s correlation coefficients among hardness, roughness, lightness, and colour change are summarized in Table 2. A strong positive correlation was observed between (hardness-lightness) (rs = 0.719, *p* < 0.001) and (lightness-color) (rs = 0.760, *p* < 0.001). Additionally, there was a moderate positive correlation between (hardness-color) (rs = 0.489, *p* < 0.001). Finally, there were strong negative correlations between (hardness-roughness) (rs=−0.855, *p* < 0.001) and (roughness-lightness) (rs=−0.573, *p* < 0.001). The correlation between (roughness-color) was not statistically significant.

**Table 2:** Correlation matrix among hardness, roughness, lightness, and colour change

Measurement	Correlation coefficient		
	Hardness	Roughness	Colour change
Roughness	−0.855*		
Colour change	0.489*	−0.451	
Lightness change	0.719*	−0.573*	0.760*

*All marked correlations are statistically significant (*p* < 0.05)

**Fig. 3:**
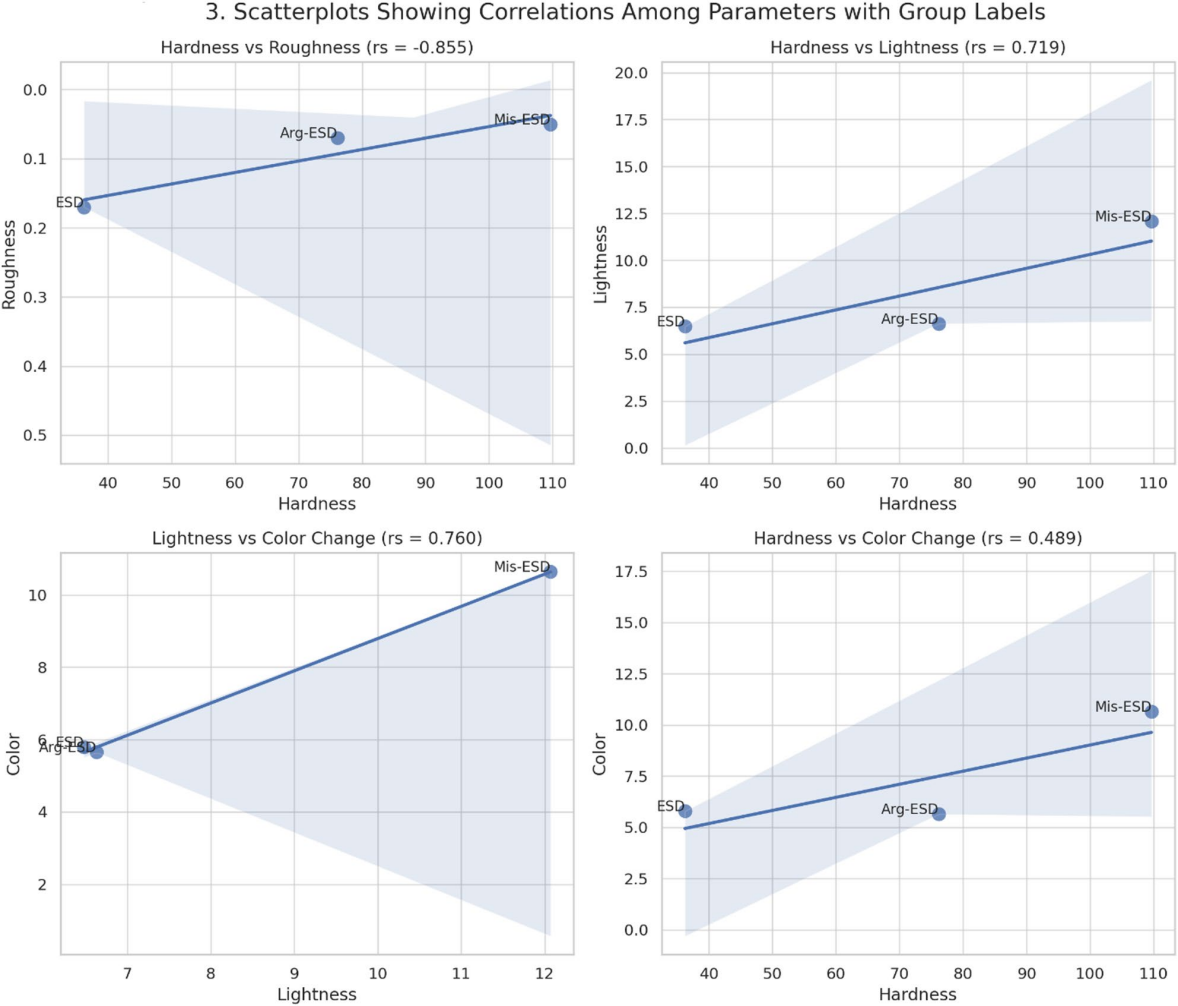
Illustrates the scatterplots demonstrating the correlations among enamel surface parameters, with each point labelled by its corresponding treatment group. (Top left: A strong negative correlation between hardness and roughness. Top right: A strong positive correlation between hardness and lightness. Bottom left: A strong positive correlation between lightness and colour change. Bottom right: A moderate positive correlation between hardness and colour change.)

Figure 3. shows a strong negative correlation between enamel hardness and surface roughness (rs = − 0.855, *p* < 0.001), meaning that specimens with higher hardness values tended to exhibit lower roughness. Conversely, hardness exhibited a strong positive correlation with lightness change (rs = 0.719, *p* < 0.001) and a moderate positive correlation with overall colour change (rs = 0.489, *p* < 0.001), suggesting that as enamel hardness improved, so did the optical properties of the surface. Furthermore, a strong positive correlation was found between lightness and colour change (rs = 0.760, *p* < 0.001), while roughness showed a significant negative correlation with lightness change (rs = − 0.573, *p* < 0.001). The correlation between roughness and colour change, however, was not statistically significant (*p* > 0.05).

These visual relationships confirm that both Arg-ESD and Mis-ESD groups contributed to enamel preservation, as evidenced by improved hardness and surface smoothness, alongside favourable changes in aesthetic parameters.

### Results of surface morphology and mineral content analysis

**Fig. 4:**
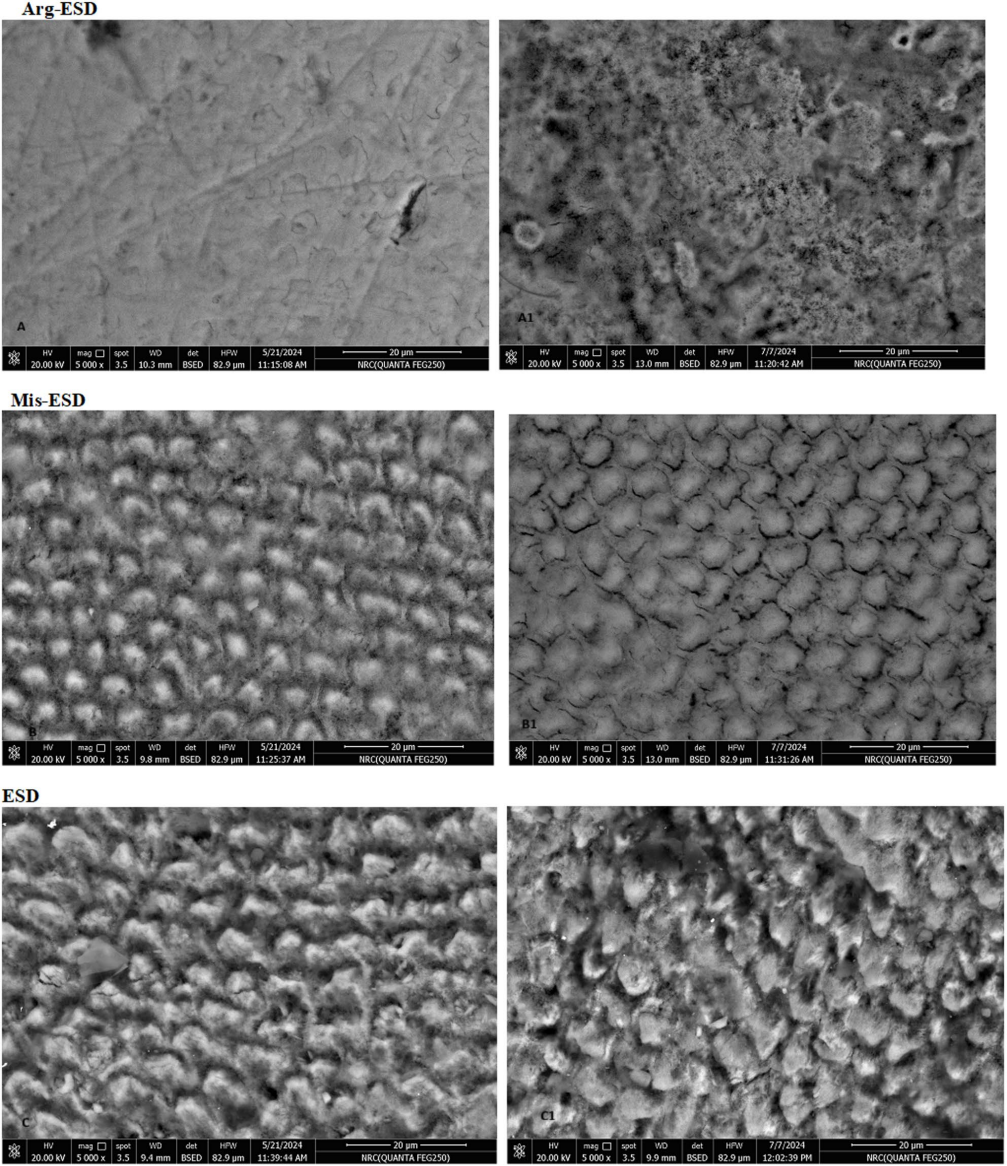
**A**, **B**, **C** SEM images ( right column) of the surface enamel discs before the start of the experiment, corresponding SEM images (left column)of the same sample (A1, B1, C1) after 7 days of immersion in its assigned solution (Arg-ESD, Mis-ESD, ESD), respectively.

Figure 4 displays the SEM images of enamel surfaces before and after 7 days of immersion in ESD, Arg-ESD, and Mis-ESD. Enamel surfaces exposed to plain ESD exhibited severe erosion with pronounced surface degradation, evident as irregular topography, porosities, and loss of surface integrity. In contrast, enamel immersed in Arg-ESD showed less extensive damage, with comparatively smoother surfaces and fewer erosive features. The Mis-ESD group demonstrated the most preserved enamel morphology, retaining surface smoothness and structure closely resembling untreated enamel, indicating a notable protective effect of Miswak extract.

**Fig. 5:**
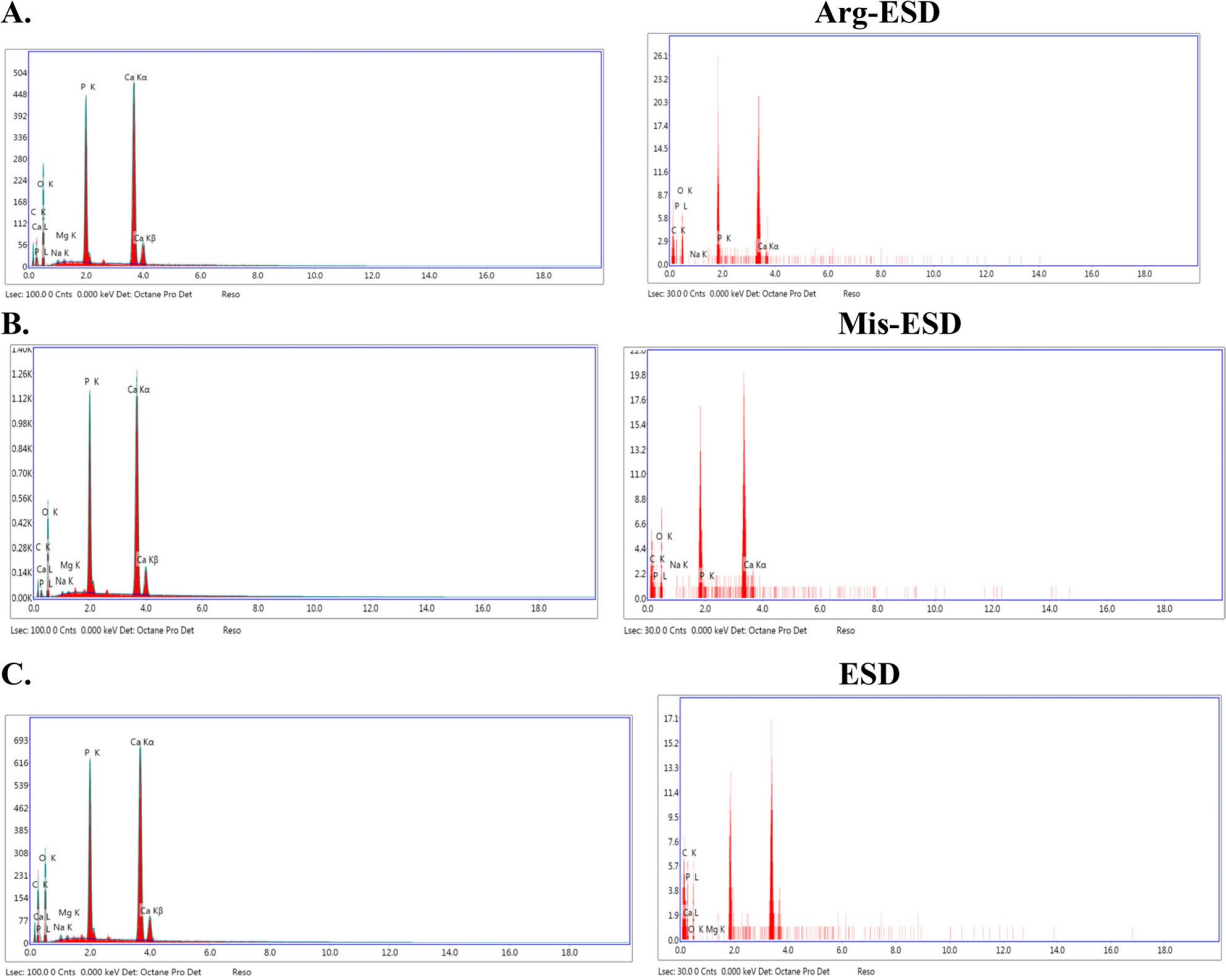
Energy-dispersive X-ray spectra collected on sound enamel (**A**, **B**, **C**) were compared to corresponding the same samples (Arg-ESD, Mis-ESD, ESD) after 7 days of immersion in its assigned solution respectively. The X-ray spectra were collected with an accelerating voltage of 15 kV

Figure 5; Table 3 present the energy-dispersive X-ray spectroscopy (EDX) results quantifying the elemental composition of the enamel surfaces pre- and post-treatment. A clear reduction in mineral content—specifically calcium (Ca) and phosphorus (P)—was observed in all groups compared to baseline, though to varying degrees:

- In the ESD group, calcium dropped to 7.57 wt% and phosphorus to 12.39 wt%, indicating substantial mineral loss. The Ca/P weight ratio was approximately 0.61, suggesting significant demineralization.
- The Arg-ESD group retained 8.29 wt% Ca and 16.24 wt% P, resulting in a Ca/P ratio of ~ 0.51, indicating that although L-arginine provided some protection, phosphorus retention was more pronounced than calcium.
- The Mis-ESD group displayed 6.65 wt% Ca and 13.51 wt% P, yielding a Ca/P ratio of ~ 0.49. Despite a slightly lower calcium retention than Arg-ESD, the enamel morphology appeared better preserved under SEM, possibly due to enhanced surface coating or buffering effects of the Miswak extract.

**Table 3:** The results mean weight % of chemical elements at the enamel surface of each sample before and after 7 days of immersion in its assigned solution

	Carbon		Oxygen		Sodium		Magnesium		Phosphorous		Calcium	
	Before	After	Before	After	Before	After	Before	After	Before	After	Before	After
Arg-ESD	8.68	29.04	41.76	43.45	0.96	2.13	0.45	0.84	15.29	16.24	32.86	8.29
Mis-ESD	2.61	36.14	40.45	38.58	0.45	2.99	0.13	2.12	18.07	13.51	38.3	6.65
ESD	21.37	46.98	34.28	29.39	0.82	2.36	0.32	1.32	13.51	12.39	29.69	7.57

In addition to calcium and phosphorus, both Arg-ESD and Mis-ESD samples showed elevated levels of magnesium and sodium, elements potentially involved in enamel stabilization and ion exchange.

Overall, the SEM and EDX findings together suggest that the incorporation of L-arginine and Miswak extract into sports drinks attenuated enamel erosion. While both agents demonstrated protective effects, Miswak extract provided more visible morphological preservation, whereas L-arginine showed slightly higher mineral retention, particularly of phosphorus.

## Discussion

Lessening the potential erosive effect of electrolyte sports drinks (ESD) on athletes’ teeth without altering the beverage’s palatability is considered a big challenge. Additionally, the direct comparison between the protective effects of L-arginine and aqueous Miswak extract against enamel erosion has not been previously explored. For these reasons, two modified ESDs formulation enclosing 1% L-arginine and 10% miswak were prepared. These precise concentrations were chosen according to previously published literature who proved the effectiveness of both concentrations [15, 16, 21, 28, 29].

The two modified ESDs (Arg-ESD and Mis-ESD) were evaluated regarding the effect of the added ingredients on the pH and taste of the original ESD.

Moreover, teeth samples were immersed in the two modified ESDs formulation as well as the original ESD for 5 min daily over 7 consecutive days at 37 °C, then the teeth samples were inspected for surface microhardness, roughness, color, morphology, and mineral content.

To assess and compare the surface roughness of tooth samples, Atomic Force Microscopy (AFM) was used. AFM is widely used in dental research due to its high resolution, versatility, and quantitative surface analysis capabilities [42]. AFM enables the direct measurement of surface roughness parameters, offering a quantitative comparison between samples [43]. It is a non-destructive technique; it doesn’t require conductive coatings thus preserve tooth integrity while analysing micro- and nano-scale surface topographic features [44].

Sa is defined as the mean of absolute height deviations from a reference plane over a 3D area (ISO 25178). Measuring Sa provides a standardized 3D quantification of surface roughness. Unlike Ra (which is 2D and linear), Sa accounts for the entire scanned area, offering a more representative measure of real surface topography [45].

As shown in the surface roughness results, tooth samples immersed in both Arg-ESD and Mis-ESD solutions showed a significantly lower surface roughness compared to those immersed in ESD, indicating that the addition of 1% L-arginine and 10% miswak effectively reduced the negative erosive effect of ESD on enamel, however, no significant difference was observed among both modified groups. The reduction in the roughness of the samples immersed in Arg-ESD and Mis-ESD solutions could be related to the difference in the pH of the three immersion solutions. The pH of ESD was very acidic both initially and after 48 h (2.93–2.97). However, addition of 1% L-arginine and 10% miswak elevated the pH of the modified formulations into (4.06–4.15) and (4.12–4.14) respectively. This elevation in pH positively affected teeth erosion though it is still below the critical threshold (~ 5.5).

It is well known that enamel is susceptible to demineralization in the pH levels between 4 and 5, especially with prolonged exposure, however, L-arginine, being capable of forming arginine-calcium-fluoride complexes [14] can positively enhance remineralization and reduce erosion.

Furthermore, *Salvadora persica* (commonly known as miswak) encloses many bioactive compounds, as fluoride [19], calcium [46], tannins [46], and flavonoids [47]. These compounds collectively contribute to the enamel remineralization ability and buffering capacity of miswak, supporting its traditional use in maintaining oral health. A study was conducted to evaluate the impact of aqueous miswak extracts on enamel surfaces subjected to erosion. Using atomic force microscopy and surface roughness analysis, the researchers found that enamel treated with miswak extract displayed improved mechanical resilience and smoother surface topography at the nanoscale, suggesting its use as a protective measure against enamel erosion and demineralization [18].

Additionally, the SEM analysis provided valuable insight into the morphological alterations of enamel surfaces following exposure to the tested drinks. Enamel immersed in plain ESD exhibited severe surface deterioration characterized by irregular topography, porosities, and apparent loss of structural integrity, confirming the aggressive erosive potential of acidic sports drinks, as previously described by Ganss et al. [48]. In contrast, enamel surfaces exposed to Arg-ESD showed notably less damage, with relatively smoother morphology and fewer erosive features. This could be attributed to L-arginine’s capacity to form protective complexes that may modulate demineralization [49]. Most notably, enamel surfaces treated with Mis-ESD retained much of their original smoothness and topography, with minimal morphological disruption, closely resembling untreated enamel. This preservation suggests a protective barrier effect likely mediated by miswak’s rich composition of bioactive and remineralizing compounds such as fluoride, calcium, and polyphenols [50–52]. These SEM findings corroborate the surface roughness and hardness data, reinforcing the idea that aqueous miswak extract provides superior morphological preservation and erosion resistance when incorporated into acidic beverages.

Regarding tooth colour after immersion in different tested solution, it was obvious that adding Miswak to ESD solution improved both tooth lightening Δ*L* and colour change Δ*E* recording the most statistically significant difference in comparison to Arg-ESD or ESD groups. The improved aesthetic appearance after using Mis-ESD modified solution might be due to its remineralizing effect and mineral content which in turn could resist the erosive process. Our results were in accordance with Nordin et al. in 2020 [53] who stated that Miswak products could enhance tooth remineralization as well as colour change. Moreover, Arg-ESD or ESD groups showed a statistically significant difference either in Δ*L* and Δ*E* while no difference was recorded between both groups. Al Taee and Al Hamdany in 2023 [54] stated that arginine containing toothpaste wasn’t effective in improving tooth color. To the best of our knowledge, no previous research has evaluated the effect of adding either Miswak or Arginine to ESDs on colour change, as most research assessed the effect of such materials on different tooth surface properties like surface hardness and roughness [14, 15, 18, 19].

## Conclusion

Within the limitations of this study, both *Salvadora persica (*Miswak) extract and L-arginine, when incorporated into electrolyte sports drinks (ESDs), demonstrated protective effects against enamel erosion. However, Miswak-enriched ESDs exhibited superior outcomes in terms of enhancing enamel surface microhardness and improving colour stability. Furthermore in-vivo taste assessments indicated acceptable taste profiles for both formulations, supporting their potential for consumer use. These findings suggest that Miswak may offer a dual benefit of erosion protection and aesthetic enhancement, making it a promising additive for formulating tooth-friendly sports beverages. Further clinical investigations are warranted to validate these effects in vivo and assess long-term benefits and consumer acceptability.

## Data Availability

The entirety of the data analysed in this study has been included in the published article. Upon making a reasonable request, the corresponding author will provide access to the raw data.

## References

[1] von Fraunhofer JA, Rogers MM. Effects of sports drinks and other beverages on dental enamel. Gen Dent. 2005;53(1):28–31. 15779219

[2] Mathew T, Casamassimo PS, Hayes JR. Relationship between sports drinks and dental erosion in 304 university athletes in Columbus, Ohio, USA. Caries Res. 2002;36(4):281–7. 12218278 10.1159/000063927

[3] Malinauskas BM, Aeby VG, Overton RF, Carpenter-Aeby T, Barber-Heidal K. A survey of energy drink consumption patterns among college students. Nutr J. 2007;6:35. 17974021 10.1186/1475-2891-6-35 PMC2206048

[4] Cochrane NJ, Yuan Y, Walker GD, Shen P, Chang CH, Reynolds C, et al. Erosive potential of sports beverages. Aust Dent J. 2012;57(3):359–64. 22924362 10.1111/j.1834-7819.2012.01708.x

[5] Pinto SC, Bandeca MC, Silva CN, Cavassim R, Borges AH, Sampaio JE. Erosive potential of energy drinks on the dentine surface. BMC Res Notes. 2013;6:67. 23422044 10.1186/1756-0500-6-67 PMC3599422

[6] Coombes JS. Sports drinks and dental erosion. Am J Dent. 2005;18(2):101–4. 15973827

[7] Huysmans MC, Chew HP, Ellwood RP. Clinical studies of dental erosion and erosive wear. Caries Res. 2011;45(Suppl 1):60–8. 21625134 10.1159/000325947

[8] Noble WH, Donovan TE, Geissberger M. Sports drinks and dental erosion. J Calif Dent Assoc. 2011;39(4):233–7. 21675676

[9] Peters MC. Strategies for noninvasive demineralized tissue repair. Dent Clin North Am. 2010;54(3):507–25. 20630193 10.1016/j.cden.2010.03.005

[10] Kui-Long LV, Yuan HW, Meng XC, Li XY. Remineralized evaluation of nano-hydroxyapatite to artificial caries. Adv Mater Res. 2010;105–106:576–9.

[11] Forzano I, Avvisato R, Varzideh F, Jankauskas SS, Cioppa A, Mone P, et al. L-Arginine in diabetes: clinical and preclinical evidence. Cardiovasc Diabetol. 2023;22(1):1–7. 37072850 10.1186/s12933-023-01827-2 PMC10114382

[12] Gonzalez AM, Townsend JR, Pinzone AG, Hoffman JR. Supplementation with nitric oxide precursors for strength performance: a review of the current literature. Nutrients. 2023;15(3):660. 36771366 10.3390/nu15030660 PMC9921013

[13] Zaura E, Twetman S. Critical appraisal of oral pre- and probiotics for caries prevention and care. Caries Res. 2019;53(5):514–26. 30947169 10.1159/000499037

[14] Bijle MNA, Ekambaram M, Lo EC, Yiu CKY. The combined enamel remineralization potential of arginine and fluoride toothpaste. J Dent. 2018;76:75–82. 29935996 10.1016/j.jdent.2018.06.009

[15] Abdslam A, Farag M, Mahfouz Omer S. Evaluation of the effect of two different concentration of arginine on fluoride uptake by demineralized enamel surfaces: in vitro study. Dental Science Updates. 2022;3(2):199–208.

[16] Elgamily HM, Aboalazm E, Safwat EM, Youssef AM. Enhancing the durability and antibacterial activity of glass ionomer restorative material enriched by l-arginine and nano-titanium for pit and fissure sealing. Discov Appl Sci. 2024;6(2):32.

[17] Almas K, Al-Bagieh N. The antimicrobial effects of bark and pulp extracts of miswak, *Salvadora persica*. Biomed Lett. 1999;60:71–5.

[18] Almas K, Al-Zeid Z. The immediate antimicrobial effect of a toothbrush and Miswak on cariogenic bacteria: a clinical study. J Contemp Dent Pract. 2004;5(1):105–14. 14973564

[19] Halawany HS. A review on Miswak (*Salvadora persica*) and its effect on various aspects of oral health. Saudi Dent J. 2012;24(2):63–9. 23960531 10.1016/j.sdentj.2011.12.004 PMC3723367

[20] Sofrata A, Lingstrom P, Baljoon M, Gustafsson A. The effect of Miswak extract on plaque pH: an in vivo study. Caries Res. 2007;41(6):451–4. 17823507 10.1159/000107931

[21] Hoobi NM, Hussein B, Qasim AA, Abdulrahman M. Dissolution of calcium ion from teeth treated with different concentrations of Siwak water extract in comparison with sodium fluoride. J Bagh Coll Dent. 2014;26:166–70.

[22] Hughes JA, West NX, Parker DM, van den Braak MH, Addy M. Effects of pH and concentration of citric, malic, and lactic acids on enamel, in vitro. J Dent. 2000;28:147–52. 10666974 10.1016/s0300-5712(99)00060-3

[23] Grenby TH. Lessening dental erosive potential by product modification. Eur J Oral Sci. 1996;104:221–8. 8804890 10.1111/j.1600-0722.1996.tb00071.x

[24] Reynolds EC. Anticariogenic complexes of amorphous calcium phosphate stabilized by casein phosphopeptides: a review. Spec Care Dentist. 1998;18:8–11. 9791302 10.1111/j.1754-4505.1998.tb01353.x

[25] Hughes JA, West NX, Parker DM, Newcombe RG, Addy M. Development and evaluation of a low erosive blackcurrant juice *in vitro* and *in situ*. J Dent. 1999;27:285–9. 10193106 10.1016/s0300-5712(98)00069-4

[26] Larsen MJ, Nyvad B. Enamel erosion by some soft drinks and orange juices relative to their pH, buffering effect, and contents of calcium phosphate. Caries Res. 1999;33:81–7. 9831784 10.1159/000016499

[27] Singh A, Masuku M. Sampling techniques & determination of sample size in applied statistics research: an overview. Int J Commer Manag. 2014;II(11):1–22.

[28] Mohamed SA, Khan JA. Antioxidant capacity of chewing stick Miswak *Salvadora persica*. BMC Complement Altern Med. 2013;13:40. 23432926 10.1186/1472-6882-13-40 PMC3607854

[29] Abhary M, Al-Hazmi AA. Antibacterial activity of Miswak (*Salvadora persica* L.) extracts on oral hygiene. J Taibah Univ Sci. 2016;10:513–20.

[30] Elbatanony MM, Safwat EM, El-Sherif S, Hassan ML. Resin-based dental pulp capping restoration enclosing silica and portlandite nanoparticles from natural resources. Sci Rep. 2024;14(1):16554. 39019960 10.1038/s41598-024-66728-0 PMC11255305

[31] Meilgaard M, Civille GV, Carr TB. Sensory evaluation techniques. 2nd ed. Boca Raton: CRC; 1991. pp. 71–4.

[32] Bergara-Almeida S, Aparecida M, Da Silva AP. Hedonic scale with reference: performance in obtaining predictive models. Food Qual Prefer. 2002;13(1):57–64.

[33] Safwat EM, Alkabani YM, Zaki DY, Elbatanony MM, Abd-Elsatar AG, Saleh RS, et al. Preparation and characterization of dental pit and fissure sealant based on calcium sodium silicate bioactive glasses. Silicon. 2023;15(16):6785–800.

[34] Eisenburger M, Addy M. Evaluation of pH and erosion time on demineralisation. Clin Oral Investig. 2011;5:108–11. 10.1007/s007840100108 11480807

[35] Barbour ME, Parker DM, Allen GC, Jandt KD. Human enamel dissolution in citric acid as a function of pH in the range 2.30 ≤ pH ≤ 6.30: a nanoindentation study. Eur J Oral Sci. 2003;111(3):258–62. 12786958 10.1034/j.1600-0722.2003.00039.x

[36] Khater GA, Safwat EM. Preparation and characterization of enstatite-leucite glass-ceramics for dental restoration. J Non-Cryst Solids. 2021;563:120810.

[37] Safwat EM, Abdel-Gawad SA, Shoeib MA, El-Hadad S. Electrochemical anodization of cast titanium alloys in oxalic acid for biomedical applications. Front Chem Sci Eng. 2024;18(1):2.

[38] Gadallah LK, Safwat EM, Saleh RS, Azab SM, Azab MM. Effect of silver diamine fluoride/potassium iodide treatment on the prevention of dental erosion in primary teeth: an in vitro study. BDJ Open. 2023;9(1):24. 37414762 10.1038/s41405-023-00153-9 PMC10325975

[39] Elgamily HM, Aboelezz A, Abdelhamid M, Youssef A. Influence of pre-treatment with diode laser and nano silica coating crosslinking matrix metalloproteinase inhibitor on the stabilization of resin-dentine interfaces. Lasers Dent Sci. 2024;8(1):30.

[40] Vilhena FV, et al. Biomimetic mechanism of action of fluoridated toothpaste containing proprietary REFIX technology on the remineralization and repair of demineralized dental tissues: an in vitro study. Eur J Dent. 2021;15(2):236–41. 33242916 10.1055/s-0040-1716781 PMC8184280

[41] R Core Team. R: A language and environment for statistical computing. R Foundation for Statistical Computing, Vienna, Austria. 2024. Available from: https://www.R-project.org/.

[42] Kumar A, Rao KM. Comparative evaluation of surface roughness of enamel following different enamel conditioning methods using atomic force microscopy. J Conserv Dent. 2011;14(2):186–92.

[43] Vahabi S, Salman BN, Javanmard A. Atomic force microscopy application in biological research: a review study. Iran J Med Sci. 2013;38(2):76–81. 23825885 PMC3700051

[44] Souza ROA, et al. Surface roughness and topography of ceramics after finishing and polishing: comparison between atomic force microscopy and contact profilometry. J Prosthet Dent. 2012;108(6):451–7.

[45] Leach R, editor. Characterisation of areal surface texture. Volume 1. Berlin: Springer; 2013.

[46] Ahmad H, Rajagopal K. Biological activities of *Salvadora persica* L. (Meswak). Med Aromat Plants. 2013;2(4):1–5.

[47] Akhtar J, Siddique KM, Bi S, Mujeeb M. A review on phytochemical and pharmacological investigations of Miswak (*Salvadora persica* Linn). J Pharm Bioallied Sci. 2011;3(1):113–7. 21430961 10.4103/0975-7406.76488 PMC3053508

[48] Ganss C, Schlechtriemen M, Klimek J. Dental erosion in children and adolescents: a cross-sectional study and a longitudinal follow-up study to detect risk factors. Caries Res. 2001;35(6):427–32.11799283

[49] Shellis RP, Featherstone JD, Lussi A. Understanding the chemistry of dental erosion. Monogr Oral Sci. 2014;25:163–79. 24993265 10.1159/000359943

[50] Al-Otaibi M, Al-Harthy M, Gustafsson A, Angmar-Månsson B. The effectiveness of chewing Miswak on plaque removal and gingival health. Saudi Dent J. 2003;15(3):135–44. 15643758

[51] Al-Bayati FA, Sulaiman KD. In vitro antimicrobial activity of *Salvadora persica* L. extracts against some isolated oral pathogens in Iraq. Turk J Biol. 2008;32(1):57–62.

[52] Al-Dosari MS, Al-Megrin WA, Al-Khedhairy AA. Chemical composition and antimicrobial activity of *Salvadora persica* L. (Miswak) extract against some pathogens. J Med Plants Res. 2012;6(1):120–5.

[53] Nordin A, Saim AB, Ramli R, Hamid AA, Nasri NWM, Idrus RBH. Miswak and oral health: an evidence-based review. Saudi J Biol Sci. 2020;27(7):1801–10. 32565699 10.1016/j.sjbs.2020.05.020 PMC7296476

[54] Al-Taee AAK, Al-Hamdany AK. A comparison of color improvement of NovaMin, casein phosphopeptide-amorphous calcium phosphate and arginine treatments applied to artificial white spot lesions. J Res Med Dent Sci. 2023;11(2). https://www.jrmds.in/articles/a-comparison-of-color-improvement-of-novamin-casein-phosphopeptideamorphous-calcium-phosphate-and-arginine-treatments-ap-100213.html.

